# Access to Health Care in Appalachia: Perception and Reality

**DOI:** 10.13023/jah.0304.10

**Published:** 2021-10-25

**Authors:** Michele Morrone, Cory E. Cronin, Kristin Schuller, Shannon E. Nicks

**Affiliations:** Ohio University, Department of Social and Public Health

**Keywords:** Appalachia, health disparities, access to care, perception of access to care

## Abstract

**Introduction:**

Health disparities such as cancer and diabetes are well documented in Appalachia. These disparities contribute to health status, and by many indicators, Appalachian people are less healthy than those who live in other parts of the country. Access to health care is one factor that contributes to health disparities. Access to care is complex and involves both intrinsic and extrinsic aspects, including satisfaction with quality of care. This research sought to compare Appalachian to non-Appalachian communities in terms of perceptions of access to care.

**Methods:**

We implemented a statewide survey to quantify perceptions of multiple components of access to care, including satisfaction with quality of care. We compared survey results to quantitative data from the County Health Rankings to document consistency with perceptions of access to care. We used chi-square analysis to compare Appalachian with non-Appalachian respondents.

**Results:**

More than 600 people completed the survey. Results of the survey identify significant differences between Appalachian and non-Appalachian residents’ perceptions of access to care and their satisfaction with health care. Specifically, Appalachian residents are less satisfied with convenience, information, quality, and courtesy of health care. They perceive providers relying on stereotypes when communicating with patients.

**Implications:**

Examining and documenting perceptions of health care is important because it could lead to improving access by focusing on cultural competency in addition to more resource intensive strategies. Health disparities in Appalachia might be minimized by being more compassionate and understanding of people who live here.

## INTRODUCTION

By many indicators, health outcomes in Appalachia are worse than other parts of the country. Numerous studies have compared health status within the 420 counties designated as Appalachian to those outside of the federally defined political boundaries. For example, the Appalachian Regional Commission found that, in 33 out of 41 health indicators, Appalachia performed worse than the national average.^1^ These indicators include those related to mental health such as depression and as well as those related to physical health conditions such as diabetes. Explanations for these health disparities include individual behaviors and those that are more systemic such as socioeconomic conditions and access to care.^2^

Addressing health disparities in Appalachia is more complicated than focusing solely on behavior change and must include attention to healthcare systems; specifically, inequities with access to care. A comprehensive definition of access to care is one component of overall health outcomes. Importantly, access cannot be defined only as proximity to health services.^3^ Cost is a major barrier to care,^4^ as is insurance availability and health literacy, especially in rural Appalachian areas.^5^

**Figure 1:**
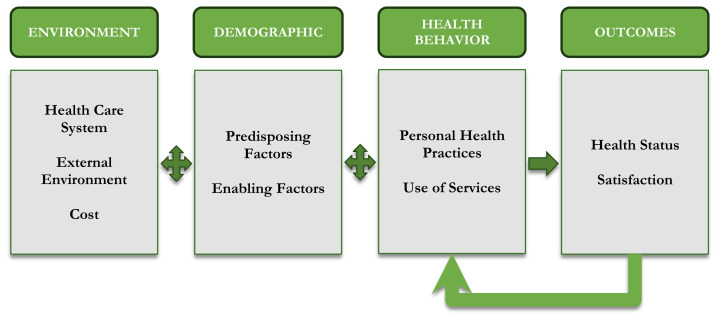
Framework to Measure Access to Healthcare, Adapted from Andersen et al.^6^

Healthcare access is complex and includes characteristics of the health delivery system, the population at risk, and how people use and are satisfied with their services. Specifically, access is related to “who people are (their individual characteristics) and where they live (community characteristics).”^6^ A framework for understanding and examining access to care is found in Figure 1. In this framework, environmental factors that affect access include location and number of providers, the cost of services, and the healthcare system. In addition, external environmental factors such as opportunities for physical activity and access to healthy foods contribute to the need to access care.

According to Andersen et al., a holistic framework for healthcare access is influenced by predisposing and enabling factors.^6^ Predisposing demographic characteristics are inherent such as age, race, and disability status, and can lead to discriminatory practices that inhibit access. In contrast, enabling characteristics are more malleable and include income, insurance, employment, and education. In many cases governmental policies and programs can temper the impact of enabling characteristics. These are vital components of a complete healthcare access framework, but perceptions of access to care and healthcare quality also play an integral role.^7^

Since the overall goal of access to health care is to improve health outcomes of individuals and populations, it is imperative that policies not focus only on capital investments in facilities or physician training, but they also promote connections between providers and their communities. Satisfaction with healthcare services and perception of access is important to understanding how community context affects access, particularly in a region where people tend to lack trust in institutions and authority.^8^–^10^ When people are satisfied with the quality of their health care and trust their providers, they are more likely to maintain relationships and use services that can improve their health. This issue is further exacerbated by historical tendencies to negatively stereotype the Appalachian population as “uneducated and dumb,” “backwards and forgotten,” and “rednecks and hillbillies.”^11^

Appalachia presents unique challenges to creating a holistic approach to improving access to care. Studies have documented disparities in health behaviors, services, and outcomes based on secondary data, but have not documented the alignment between this data and how people perceive their access. Considering the multi-faceted nature of healthcare access, perception may be at least as important as health behaviors and presence of clinics and physicians. As such, we were interested in exploring the relationship between perception and access to care in Appalachia. Our two questions of interest are:

1. Is there consistency between perceptions of access to care and quantitative indicators derived from secondary data sources?
2. Are there differences between Appalachian and non-Appalachian residents in terms of qualitative measures of health outcomes relevant to healthcare access?

The first question generally seeks to validate perceptions that people have about healthcare access where they live. Findings from the first question provide some basis on which to make the argument that people who live in Appalachia understand the conditions with access to care in their communities. This gives some weight to answering the second question. If perception of healthcare access is consistent with quantitative data, then perhaps qualitative satisfaction should be considered as a key factor in improving this access.

## METHODS

Using the framework for access to care summarized in Figure 1, an online survey in Ohio was employed to gather primary data about how residents view access to care. Initially, the researchers worked with a local group of healthcare professionals in one Appalachian County to create the survey. This group was tasked with improving healthcare access as part of their Community Health Improvement Plan. Multiple survey drafts were developed and, prior to finalizing the survey, two focus groups were held. The focus groups helped to refine the survey further. After finalizing the survey, it was mailed to a random list of people who reside in this one county, similar to a pilot test for the statewide effort. Data from the pilot survey indicated that it is a valid tool for assessing perceptions about healthcare access. The survey was approved by the Institutional Review Board at Ohio University and was live for three months at the end of 2018.

Respondents identified their county of residence, allowing us to compare those who live within the Appalachian region of the state, as defined by the Appalachian Regional Commission, with those who do not. Of the 88 counties in Ohio, 32 of these (in the eastern and southeastern part of the state) are within the political boundaries of Appalachia.

To answer the question about whether perception is consistent with documented conditions, Ohio county-level data were gathered from the 2019 County Health Rankings (CHR), curated by the University of Wisconsin Population Health Institute and supported by the Robert Wood Johnson Foundation.^12^ The CHR uses secondary data from sources such as the American Community Survey, the Behavioral Risk Factor Surveillance System, the National Center for Health Statistics, and others to score each county on health outcomes and health factors. This score is then used to compare counties within and among states.

Two distinct data sets were used: results from a statewide survey and data from a national database. Four indicators were selected to represent the environment, demographics, and health behaviors (2) in the framework in Figure 1. The environment component of the framework includes the healthcare system so, for this indicator, the percentage of survey respondents who think there are enough services in their county was compared to the data compiled in the CHR for the rate of primary care providers per 100,000 population. The enabling demographic factor is the percent uninsured as self-identified in the survey and the percent uninsured in the CHR.

Two indicators were used for health behaviors. One was the percentage from the survey who said they used preventive screening as compared to the percent in the CHR that had a mammogram, since the CHR does not document general preventive screening. Finally, the indicator for personal health practices was the percent of survey respondents who used recreation/wellness facilities and the percent from the CHR that have access to exercise opportunities. For each indicator, statistically significant differences between Appalachian and non-Appalachian counties were evaluated; since the CHR data is continuous, the counties were categorized into quartiles to use chi-square.

For the second research question, perceptions about access to care were identified, specifically in terms of satisfaction, which is one component of health outcomes in our overall model of access (Figure 1). To compare health outcomes between Appalachian and non-Appalachian respondents, survey data was used that asked respondents to identify their level of satisfaction in the convenience, cost, quality, information, and courtesy of providers. No definitions were provided for these five factors, instead relying on the respondent to self-define when rating their satisfaction on a 3-point scale (satisfied, somewhat satisfied, or not satisfied at all). Pearson chi-square tests were used to identify statistically significant differences between Appalachian and non-Appalachian respondents. The survey also provided an opportunity for respondents to include open-ended comments in response to a prompt, “Use the space below to write comments, questions, and ideas that you would like to share with healthcare providers.” These responses were reviewed to identify concerns specifically related to healthcare provider satisfaction indicators noted above.

## RESULTS

Because of the recruiting strategy, it is not possible to calculate a response rate for the survey. Of the 695 people who responded to the survey, 438 (63.4%) identified themselves as residents of one of the 32 Appalachian counties in the state. Regardless of county, most of the respondents were women (Appalachian: 82%; non-Appalachian: 72.8%). Appalachian respondents were older than non-Appalachian respondents; the average age of Appalachian respondents was 69.15 and only 48.76 for those not in Appalachian Ohio. Survey responses were compared for four factors in the healthcare access framework with indicators from the County Health Rankings. These are summarized in Table 1.

### Comparisons of Healthcare Access Factors

There are fewer healthcare professionals in Appalachian Ohio counties than non-Appalachian counties; both the survey data and the County Health Rankings data demonstrate this. Only 29% of the survey respondents who live in Appalachian counties think there are enough healthcare services in their county, compared to 57% of the respondents from non-Appalachian counties. This is consistent with the difference in services reported by CHR, which identifies the rate of primary care physicians to people as 60% lower in Appalachian than non-Appalachian Ohio counties. However, the difference in the survey data between Appalachian and non-Appalachian respondents is statistically significant while the difference in the CHR data is not.

One individual enabling factor related to access is insurance coverage. Table 1 compares self-reported insurance coverage between Appalachian and non-Appalachian survey respondents with data from the CHR. Survey respondents from Appalachia are more likely to say they are uninsured than those who are not in the region, and the quantitative data support this perception. Both the survey data and the CHR data indicate that there are significant differences in insurance coverage between Appalachian and non-Appalachian counties.

There are several indicators of health behaviors in the access to care framework. Two components relevant to this research are personal health practices and how people use health care including the rate of preventive screening. As Table 1 shows, less than one-fourth of the Appalachian survey respondents say they used preventive screening services, with even fewer accessing these services in their home counties (12.8%). Though the CHR report a higher percentage of screening services than the survey data, it is still lower than non-Appalachian counties.

Physical activity is a component of personal health practices in the access framework. Only 17.6% of Appalachian survey respondents said they used recreational/wellness resources such as exercise classes in the previous 12 months and 22.1% of respondents in non-Appalachian counties did. So, while use of services is similar, the difference is that about three-fourths of Appalachian respondents who accessed these services did so in their home county and all of the non-Appalachian respondents who accessed these resources stayed in their home counties to do so. According to County Health Rankings, 59% of Appalachia has access to exercise opportunities compared to 73% in non-Appalachian counties.

### Health Outcomes: Satisfaction

Novel data from the survey are measures of how satisfied people are with the services they receive. As Table 2 shows, respondents from Appalachia are significantly less satisfied with healthcare services than those outside of the region, with one exception: perceptions related to cost are similar regardless of county of residence. Appalachian respondents are significantly less satisfied with convenience and quality of care as well as the information from their providers.

There were 133 comments in the open-ended prompt of the survey and 94 of these, or 71% were from people in Appalachian counties. Of the 438 respondents who lived in Appalachian counties, 94 (21%) provided written comments, while 39 of the 253 non-Appalachian respondents (15%) did so. Many of these comments are directly related to satisfaction with services based on the five categories of satisfaction: courtesy, cost, convenience, information, and quality. Comments related to cost, convenience, information, and quality were similar in terms of content regardless of county of residence. Interestingly, comments relating to courtesy of providers were more prominent from Appalachian respondents. There was only one comment in the non-Appalachian subsample about provider courtesy. On the other hand, as the examples below indicate, there are strong, specific comments about the courtesy of providers from Appalachian respondents. Some of these comments directly address stereotypes previously discussed.

### Appalachian Comments Related to Courtesy

*If a patient requests a referral out of county it should be honored. We have the right to be seen where we feel most comfortable and safe. Sometimes it feels like the doctors in the area don’t care because we are dumb hillbillies. I can assure you we are not.*

*A former doctor looked at me while in gown on table, with nurse present and stated, “She’s welfare trash” and went on to criticize my financials.*

*Your “bedside manners” are important. Hasn’t happened to me, but others have shared that they are intimidated by the attitudes of doctors.*

*I went to my PCP and was told “I don’t typically see people with insurance.” This is definitely not a comment I feel like a healthcare professional should be making. She was implying that everyone else typically uses Medicaid or something of that sort.*

*I have a primary care physician, but my physician is not responsive or dismissive of some of my health issues.*

*My daughter has Medicaid for her and her children…HOWEVER, she has had multiple experiences with providers (doctors and pharmacists and pediatricians) who lack respect, are judgmental, and some are just plain rude. Insinuating that she was not taking proper care of the grandchild who had the flu. One doctor had her in tears… My daughter, while on assistance, is not an idiot. She is a human being.*

### Non-Appalachian Comment Related to Courtesy

*Work on customer service. I have changed primary care doctors simply because of the customer service. Staff should be friendly and nonjudgmental. Do not assume how much knowledge I have.*

## IMPLICATIONS

The purpose of this study was to explore the perception of access to care in Appalachia. The findings address the two research questions: (1) perception of access to health care in Appalachia is consistent with secondary data; and (2) survey respondents who reside in Appalachia Ohio are less satisfied with their healthcare services than others, especially in terms of provider courtesy.

Appalachian residents understand the limitations they face in accessing health care. When asked to share their opinions, they state that there are not enough providers, specifically in specialty care (Table 1). Most of the factors that contribute to comprehensive access to care are worse in the counties that comprise Appalachian Ohio than other counties in Ohio. This includes those community enabling factors that are not highlighted here such as poverty and unemployment. When comparing perceptions of survey respondents to secondary data, there are similarities between what people think about healthcare access where they live and what the data show.

The connection between perception and reality provides an important and validating foundation to understanding how satisfaction influences access to care. These perceptions are not as easily documented in census data, national health surveys, or other prominent sources of health data. This is the most compelling finding of this research, especially since there are stark differences between Appalachian and non-Appalachian respondents when it comes to courtesy of providers.

There are several limitations to this research. First, the snowball approach to recruiting participants include a range of potential biases.^13^ Because of the way the sample was derived, it is not possible to draw conclusions to Appalachia in general and the sample may not be representative of Appalachian Ohio. However, the perceptions related to courtesy of providers could be an important factor to address in improving overall access to care in Appalachia.

Second, there are limitations with the County Health Rankings data. These limitations include difficulty modeling population health, determining statistically significant differences between close rankings, difficulty measuring changes year over year, and reliability of data among smaller counties.^14^ Nevertheless, the CHR compile a vast amount of health data at the county level allowing researchers to compare counties based on specific characteristics (i.e., Appalachian vs. non-Appalachian).

In conclusion, when policymakers discuss how to improve access to care, the focus is often on building new facilities or training more healthcare professionals, which necessitate extensive time and resources.^6^ While policymakers and health system leaders should be attentive to the disparities that can be addressed by committing resources to healthcare infrastructure, the findings presented here suggest there might be additional ways to improve patient satisfaction and, in turn, promote greater healthcare access. Perhaps a focus on training healthcare providers to be more courteous and culturally sensitive in their day-to-day interactions with patients could provide an opportunity to lessen the healthcare access gap that exists in Appalachia.

A greater awareness of a sense of place, and of what that place means to the people who live there, would improve provider–patient relations. If people believe their providers respect and listen to them, regardless of where they live, they are more likely to be satisfied with their overall care. When they are more satisfied, they might seek care when needed, including for preventive care. Health disparities exist in Appalachia, but maybe these disparities can be mitigated, even a little bit, by simply being more compassionate and understanding of the people who live here.

**Summary Box**

**What is already known about this topic?**

Health disparities are documented in Appalachia compared to the rest of the country.

**What is added by this report?**

There is little information documenting the impact of perception of satisfaction of healthcare access. We compare perceptions of access to care with data and document differences in perceptions between Appalachian and non-Appalachian residents.

**What are the implications for future research?**

Documenting perceptions, specifically satisfaction with health, can contribute to improving access to care by focusing on raising cultural awareness among providers.

## Figures and Tables

**Table 1:** Comparisons Between Appalachian and Non-Appalachian Counties For Select Indicators of Access to Health Care

Survey				County Health Rankings (CHR)			

Opinion	Appalachian (%)	Non-Appalachian (%)	Chi-square (Sig)	Indicator	Appalachian (%)	Non-Appalachian (%)	Chi-square (Sig)*

Environmental Factor: Health System							

Percentage of respondents who think there are enough services in their county.	29	57	49.10 (0.000)	Primary care provider rate (per 100,000)	41.8	55.7	6.679 (0.083)

Demographic Factor: Individual Enabling, Insurance							

Percentage of respondents who did not have insurance at some point during the past 12 months.	7.8	3.6	4.55 (0.033)	Percent uninsured	8.3	6.8	27.11 (0.000)

Health Behaviors: Use of Services							

Percentage that used preventive screening services (in their home county)	24%	26.5	0.540 (0.462)	Annual mammogram	38.7	42.7	22.66 (0.001)
	(12.8)	(19.4)	(5.392) (0.020)				

Health Behaviors: Personal Health Practices							

Percentage that used recreation/wellness facilities (in their home county)	17.6	22.1	2.140 (0.143)	% with access to exercise opportunities	59	72.7	18.46 (0.000)
	(13.7)	(22.9)	(9.640) (0.002)				

* Chi square computed using quartiles for CHR data

**Table 2:** Comparisons of Perceptions Between Appalachian and Non-Appalachian Survey Respondents

	Appalachian n(%)	Non-Appalachian n(%)	Chi-square	Significance
**CONVENIENCE**				
Satisfied	163 (42.2)	153 (70.2)	45.95	0.000
Somewhat satisfied	150 (38.9)	51 (23.4)		
Not satisfied at all	69 (17.9)	13 (6.0)		
**COST**				
Satisfied	95 (24.7)	68 (31.3)	6.93	0.074
Somewhat satisfied	133 (34.5)	83 (38.2)		
Not satisfied at all	143 (37.1)	61 (28.1)		
**QUALITY**				
Satisfied	178 (46.2)	147 (68.4)	30.35	0.000
Somewhat satisfied	166 (43.1)	61 (28.4)		
Not satisfied at all	36 (9.4)	7 (3.3		
**INFORMATION**				
Satisfied	181 (47.0)	127 (58.8)	16.16	0.001
Somewhat satisfied	158 (41.0)	79 (36.6)		
Not satisfied at all	39 (10.1)	5 (2.3)		
**COURTESY**				
Satisfied	223 (58.1)	153 (72.2)	15.20	0.002
Somewhat satisfied	133 (34.6)	55 (25.5)		
Not satisfied at all	21 (5.5)	5 (2.3)		
